# Incidence and predictors of surgical site infection following cesarean section in North-west Ethiopia: a prospective cohort study

**DOI:** 10.1186/s12879-020-05640-0

**Published:** 2020-11-30

**Authors:** Daniel Bekele Ketema, Fasil Wagnew, Moges Agazhe Assemie, Aster Ferede, Alehegn Aderaw Alamneh, Cheru Tesema Leshargie, Getiye Dejenu Kibret, Pammla Petrucka, Animut Takele Telayneh, Animut Alebel

**Affiliations:** 1 Department of Public Health, College of Health Science, Debre Markos University, P.O. Box 269, Debre Markos, Ethiopia; 2 Department of Nursing, College of Health Science, Debre Markos University, Debre Markos, Ethiopia; 3 Faculty of Health, University of Technology Sydney, Sydney, Australia; 4 Department of Human Nutrition and Food Science, College of Health Science, Debre Markos University, Debre Markos, Ethiopia; 5 College of Nursing, University of Saskatchewan, Saskatoon, Canada; 6 School of Life Sciences and Bioengineering, Nelson Mandela African Institute of Science and Technology, Arusha, Tanzania

**Keywords:** Incidence, Surgical site infection, Caesarean section, Predictors

## Abstract

**Background:**

Following delivery by caesarean section, surgical site infection is the most common infectious complication. Despite a large number of caesarean sections performed at Debre Markos Referral Hospital, there was no study documenting the incidence of surgical site infection after caesarean section. Therefore, this study aimed to estimate the incidence of surgical site infection following caesarean section at Debre-Markos Referral Hospital in Amhara region, North-west Ethiopia.

**Methods:**

A prospective cohort study was conducted among 520 pregnant women who had a caesarean section between March 28, 2019 and August 31, 2019. Preoperative, intraoperative, and postoperative data were collected using a standardized questionnaire. Data was entered using EpiData™ Entry Version 4.1 software and analyzed using R Version 3.6.1 software. A descriptive analysis was conducted using tables, interquartile ranges and median. The time to development of surgical site infection was estimated using Kaplan-Meier method. The Cox regression model for bivariable and multivariable analyses was done. Adjusted Hazard Ratio (AHR) with 95% Confidence Interval (CI) was reported to show the strength of association.

**Result:**

The mean age of the study cohort was 27.4 ± 4.8 years. The overall cumulative incidence of surgical site infection was 25.4% with an incidence of 11.7 (95% CI:9.8,13.9) per 1000 person/days. Not able to read and write (AHR = 1.30,95% CI:1.19,2.11), no antenatal care (AHR = 2.16, 95%CI:1.05,4.53), previous history of CS (AHR = 1.21, 95% CI:1.11,2.31), HIV positive (AHR = 1.39, 95% CI:1.21,2.57), emergency procedure (AHR = 1.13, 95% CI:1.11,2.43), vertical type of incision (AHR = 2.60, 95% CI:1.05,6.44), rupture of membrane (AHR = 1.50, 95% CI:1.31,1.64), multiple vaginal examination (AHR = 1.88, 95% CI: 1.71, 3.20) were significant predictors of surgical site infection in this study.

**Conclusion:**

This study concluded that the incidence of surgical site infection following caesarean section was relatively high compared to previous studies. Not able to read and write, have no ante natal care, previous history of caesarean section, HIV, emergency surgery, vertical type of incision, rupture of membranes before caesarean section, and multiple vaginal examinations were significant predictors of surgical site infection in this study. Therefore, intervention programs should focus on and address the identified factors to minimize and prevent the infection rate after caesarean section.

**Supplementary Information:**

The online version contains supplementary material available at 10.1186/s12879-020-05640-0.

## Background

Caesarean section (CS) is a life-saving surgical procedure in high-income and low-income countries with a global range of 6 to 27.2% [1, 2]. The rate of CS in Ethiopia ranged from 1.5 to 21.8% [3]. CS can be a life-saving method and it averts poor obstetric outcomes. However, the use of CS without medical needs can put women at risk of short-term and long-term health problems [3–5].

A surgical site infection (SSI) an infection that happens at the incision/operative site (including drains) within 30 days of the post-surgical procedure [6]. SSI is a healthcare-associated infection, especially in low-income countries including Ethiopia [7–9], with reported rates ranging from 3 to 15% [10, 11]. Despite the fact that improvements have been made in sterilization methods, operating room ventilation, surgical technique, and accessibility of antimicrobial prophylaxis; SSI following CS delivery remains a significant cause of maternal illness, extended hospitalization, increased medical costs, and maternal death [9, 12–17].

Due to the continuous increment of the rate of delivery by CS; the number of women with surgical site infection is anticipated to be increased. Facility-based studies conducted in Ethiopia reported that approximately 11% of the women who had CS delivery developed SSI [18, 19]. Several studies revealed that the majority of SSIs occurred within 30 days of cesarean section and most often between the 5th and the 10th postoperative days [20, 21]. .

Different scientific literatures reported that the incidence of SSI following CS delivery depends on many factors including: wound class, maternal age, hypertensive disorders, types of CS procedures, number of vaginal examinations, high volume of blood loss during surgery, diabetes, maternal weight, surgical techniques and premature rupture of membrane [5, 9, 10, 20, 22–32].

While the above studies provide valuable information about the incidence of SSI; the evidence obtained from these studies are not generalizable since these studies were conducted in different countries with significant differences in the operating room and availability of skilled personnel. Moreover, two studies were conducted in Ethiopia on the incidence of SSI [18, 19]. A research conducted at Hawassa University Teaching and Referral Hospital, Ethiopia [18] have used cross-sectional design and the authors reported that there is a considerable limitation since the study did not evaluate many potential risk factors of SSI: like underling maternal medical condition, and skin closure method used which are addressed in this current study. The other study carried out at Jimma University Specialized Hospital, Southwest Ethiopia [19], focused on the composite incidence of SSI among women who underwent a hysterectomy, CS and destructive delivery which is difficult for focused intervention. Hence, our study focuses on the incidence of SSI following single surgical intervention (CS) which will important to intervene on amenable predictors.

Moreover, a large number of CS have been performed at Debre Markos Referral Hospital since it is the only referral hospital in Debre Markos town. As a result, there was a relatively common occurrence of SSI following CS. However, there is no study documenting the incidence of SSI after CS delivery. Hence, this study was conducted to estimate the incidence and predictors of SSI among women undergoing CS at Debre-Markos Referral Hospital. Thus, identifying the predictors of SSI helps to formulate an ideal environment and reduce SSI and its consequences. Furthermore, the results of this study necessary to develop an evidence-based preventive protocol for post-CS SSI in this clinical setting and other settings with similar contests.

## Methods

### Study setting, design, period and population

A prospective cohort study was conducted among pregnant women who had a caesarean section between March 28, 2019, and August 31, 2019, at Debre Markos Referral Hospital (DMRH). DMRH located, in the East Goiiam zone, Amhara regional state of Ethiopia. It is one of the centers where pregnant mothers can receive CS services. It provides services with three units (labor, maternity, and gynecology). It also has outpatient clinics (antenatal care, family planning). All pregnant women admitted to the DMRH labor ward who underwent an elective or emergency CS were eligible for enrolment within 24-h post-CS and followed for 30 days to detect SSI, in accordance with the Centers for Disease Control and Prevention (CDC) Classification [6]. Pregnant women who were died during the surgical procedure or immediately after the procedure or surgical procedures performed outside DMRH were excluded from the study.

### Sample size, and sampling procedure

The sample size was computed using R statistical software Version 3.6.10 based on the sample size determination formula for survival analysis. For the first objective, the required sample size was determined by considering the following assumptions based on the single population proportion. Sample size (n) is calculated by taking the proportion (P) 10.9% obtained from similar published articles in Tanzania [24], 95% confidence level, 5% margin of error (d) and used a single population proportion formula: $n=\frac{{\left( Z\alpha {/}_2\right)}^2\Big(p\left(1-p\right)}{d^2}$. This calculation yielded a N_1_ = 150.

For the second objective, the sample size was calculated using Stata™ Version 14.1 by considering two-tailed significant level (α) of 0.05 and power 80%, and a 95% confidence interval

$$ e=\frac{\left(Z\frac{a}{2}+ Z\beta \right)\hat{\mkern6mu} 2}{(lnHR)\hat{\mkern6mu} 2}\kern2em N=\frac{e}{p(e)} $$

Where e = required number of the event, p = expected cumulative incidence of SSI following CS based on the previous studies, N = total sample size, 5% non-response rate. This calculation yielded a sample size of 513 participants. Accordingly, it is recommended to use the largest sample size obtained out of all calculations in order to accommodate all study objectives. Therefore, 513 individuals were required for the study. Patients were serially enrolled until the desired sample size was reached.

**Table:** 

**Variables**	**Hazard Ratio**	**Event**	**P(e)**	**Sample size**
Hypertension disorder	2.9	7	0.083	86 [24]
Prolonged duration of labor	2.73	8	0.02	400 [24]
Duration of procedure	1.83	22	0.045	489

### Variables of the study

The dependent variable of this study was the incidence of SSIs following CS. The independent variables were socio-demographic variables like maternal age, marital status, residence, maternal educational status, occupational status, religion, and antenatal care. Relevant maternal medical history like diabetes mellitus, renal disease, anemia, HIV, bronchial asthma, previous history of CS, and/or hypertensive disorder were captured. Surgical intervention related variables were considered such as types of CS (elective or emergency), types of incision (vertical, horizontal), types of skin suturing (interrupted, subcuticular), premature rupture of membranes, number of peravaginal examination, blood loss, duration of procedures, anesthetic techniques (general, epidural), indication for CS, gestational age, blood transfusion, and/or antibiotic used.

### Data collection tools, procedures and quality control

Data were collected using a structured questionnaire that was recorded at each follow-up visit. Training on the objectives of the study and how to diagnose SSI clinically was given for data collectors and supervisors for 2 days. In addition to the patient interviews, baseline information was also retrieved from a variety of sources: medical records, antenatal cards, surgical notes, structured interviews, and clinical examinations [33]. On the first day after CS during the inpatient stay, all data related to the surgical procedure and post-surgical management were mined from the surgical notes. Demographic and clinical data were obtained from antenatal cards, medical records, structured interviews, and clinical examinations. The ward health care provider inspected the wounds daily and checked the patients for any signs of SSI during the inpatient stay. The women were followed for 30 days to detect SSI in accordance with the Centers for Disease Control and Prevention (CDC) Classification. Patients who were missed the appointed scheduled were contacted by telephone in order to determine whether they had SSI or not. A patient was considered lost to follow-up after five unsuccessful attempts to reach them by telephone during the follow-up period.

### Operational definition

Patients were considered to have SSI following CS if they met the following definition: Involving skin and subcutaneous tissue at surgical site with any one of the following: purulent discharge or organisms isolated from fluid/tissues of superficial incision or at least one sign of inflammation (pain, fever, localized swelling, induration, dehiscence, overlying skin changes and exudative purulent discharge) or wound deliberately opened by the surgeon for drainage or surgeon declares that the wound is infected [6].

#### Event

Development of SSIs following CS.

#### Time to SSI

Time (in days), between the end of operation to the development of SSI following CS.

#### Censoring

Patients not developing SSI, and those lost follow up.

#### Hemoglobin

Less than12 grams/deciliter (g/dL) was considered as the cut off point for the diagnosis of anemia.

#### Duration of operative procedure

Interval (in hours and minutes) between the CS Start Time, and the CS Finish Time.

### Data processing and analysis

Data were cleaned, coded, and entered into EpiData™ Entry software Version 4.1 and exported into R version 3.61 for analysis. A descriptive analysis of categorical variables was performed through frequency tables, and interquartile ranges and medians were computed for continuous variables. The time for the development of SSI was estimated using the Kaplan-Meier (KM) method. The log-rank test was utilized to compare the estimated survival curve of patients based on categorical variables. The proportional hazard assumption (PHA) was checked using graphs and scaled Schoenfeld residuals tests*.* Both the graph and figure suggested that there is no evidence of non-proportional hazards for the remaining covariates (S1 Table and S2 Fig). Variables with *p*-values less than 0.2 at bi-variable analysis were screened for multi-variable Cox regression model. The goodness of fit was checked by the Cox Snell residual test. The final results were taken as significant at *P* < 0.05. Adjusted Hazard Ratio (AHR) with 95% Confidence Interval (CI) were used to report strength of the association between outcome variable and predictors.

## Results

### Socio-demographic and operational related characteristics

About 520 pregnant women were undertook CS delivery between March 28, 2019 and August 31, 2019. All of these 520 pregnant women were serially enrolled and followed for 30 days to detect the development of SSI. Of these 520 CS, 422 (81.16%) were categorized as emergency CS procedures. A majority of the respondents,488 (93.8%) had ante-natal care (ANC) with a median of 4 visits (IQR:3–4) (Table 1). The age of the study subjects was ranged from 18 to 49 years with a mean age of 27.4 ± 4.8 years. The gestational age at caesarean delivery ranged between 30 and 44 weeks and a total of 485 (93.3%) neonates were delivered at term gestational age with the remaining cases delivered as 14 (2.7%) post-term and 21(4%) pre-term neonates (Table 2).

**Table 1:** Socio-demographic characteristics of patients undergoing caesarean section in Debre Markos Referral Hospital between March 28, 2019 and August 31, 2019

Variables	Category	Frequency	Proportion (%)
**Residence**	Urban	343	65.96
	Rural	177	34.04
**Religion**	Orthodox	506	93.31
	Muslim	13	2.50
	Protestant	1	0.20
**Occupation**	Housewife	294	56.5
	Merchant	67	12.9
	Government employee	129	24.80
	Student	6	1.20
	Farmer	24	4.60
**Education**	Not educated	181	34.9
	Able to read and write	51	9.81
	Primary	44	8.50
	Secondary	102	19.70
	Tertiary and above	140	27.00
**Antenatal check-up**	Yes	488	93.80
	No	32	6.20
**Place of ANC-follow up**	DMRH	192	36.92
	Outside	328	63.08
**History of CS**	Yes	413	79.43
	No	107	20.57

**Table 2:** Clinical, Obstetric and Operational characteristics of patients undergoing caesarean section in Debre Markos Referral Hospital between March 28, 2019 and August 31, 2019

Variables	Category	Frequency	Proportion (%)
**Onset of labor**	Spontaneous	400	76.9
	Induced	17	3.3
	No labor	103	19.8
**Rupture of membrane before CS**^**a**^	Yes	158	31.40
	No	362	69.60
**Per vaginal examination**	Yes	107	20.57
	No	413	79.43
**Preoperative blood transfusion**	Yes	19	3.60
	No	501	96.40
**Types of CS**^**a**^	Elective	98	18.84
	Emergency	422	81.16
**Types of incision**	Horizontal	498	95.77
	Vertical	22	4.23
**Types of skin suturing**	Interrupted	37	7.1
	Subcuticular	483	92.9
**Anesthetic techniques**	General	20	3.80
	Epidural	4	0.80
	Spinal	496	95.40
**Blood transfusion**	Not transfused	504	97.0
	1-unit	8	1.5
	2-units	8	1.5
**Surgical procedure by**	Gynecologist	453	86.9
	Emergency surgeon	65	12.5
	Junior surgeon	3	0.6
**Gestational age in weeks**	Pre-term	21	4
	Term	485	93.3
	Post-term	14	2.7
**Parity**	Nulliparous	267	51.3
	1–3	184	35.4
	≥4	69	13.3
**Number of prior CS**^**a**^	0	413	79.4
	1	81	16.7
	2	16	3.1
	3	4	0.8
**HIV Status**	Positive	7	1.3
	Negative	513	98.7
**Hypertension (Any type)**	Yes	39	7.5
	No	481	92.5

^a^cesarean section

All pregnant women had received pre-operative antibiotic prophylaxis before undergoing CS. Ceftriaxone is the most prescribed antibiotic prophylaxis (91.9%) followed by ampicillin (2.7%).

In this study, 183 (35.2%) pregnant women underwent CS with an indication of fetal distress (Table 3). The mean gestational age of the study participants who were developed SSI was 39.6 weeks (SD ± 4.62). The median follow-up time is different among women with SSI and without (8 days’ vs 29 days) (Table 4).

**Table 3:** Indication of caesarean section of patients undergoing caesarean section in Debre Markos Referral Hospital between March 28, 2019 and August 31, 2019

Indication for CS^**a**^	Frequency	Percent (%)
Fetal distress	183	35.20
Previous CS^**a**^	100	19.23
Mal presentation	93	17.88
Poor progress	13	2.500
Placenta Previa Type 3	5	1.00
Severe macrosomia	10	1.9
Multiple birth	15	2.94
Severe pre-eclampsia	9	1.73
CPD^b^	63	12.11
Uterine rupture	7	1.35
Failed induction	19	3.65
Other^**c**^	3	0.51

^a^ Caesarean section

^b^ Cephalo-Pelvic Disproportion

^**c**^ Includes cervical arrest, and ante-partum hemorrhage

**Table 4:** Summary of continuous variables of patients undergoing caesarean section in Debre Markos Referral Hospital between March 28, 2019 and August 31, 2019

Variable	Surgical site infection following CS	
	Yes	No
Age	27.6 ± 5.3	27.4 ± 4.7
Number of antenatal care (median (IQR))	4.0 (4.0,4.0)	4.0 (3.0,4.0)
Maternal weight	62.1 ± 8.8	62.0 ± 8.86
Gestational age	39.9 ± 4.62	39.6 ± 3.4
Follow up time (median (IQR))	8.0 (5.0, 13.0)	29.0 (22.0, 30.0)

*CS* Caesarean Section, *IQR* sInter Quartile Range

### Incidence of SSI following CS

**Fig. 1:**
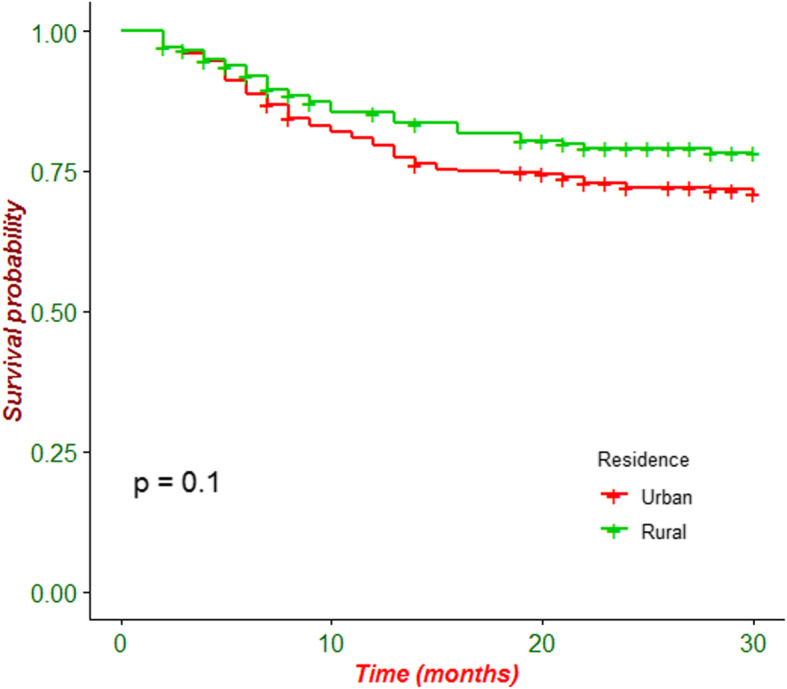
Kaplan-Meier survival curve of patients undergoing caesarean section in Debre Markos Referral Hospital between March 28, 2019 and August 31, 2019 by Residence

**Fig. 2:**
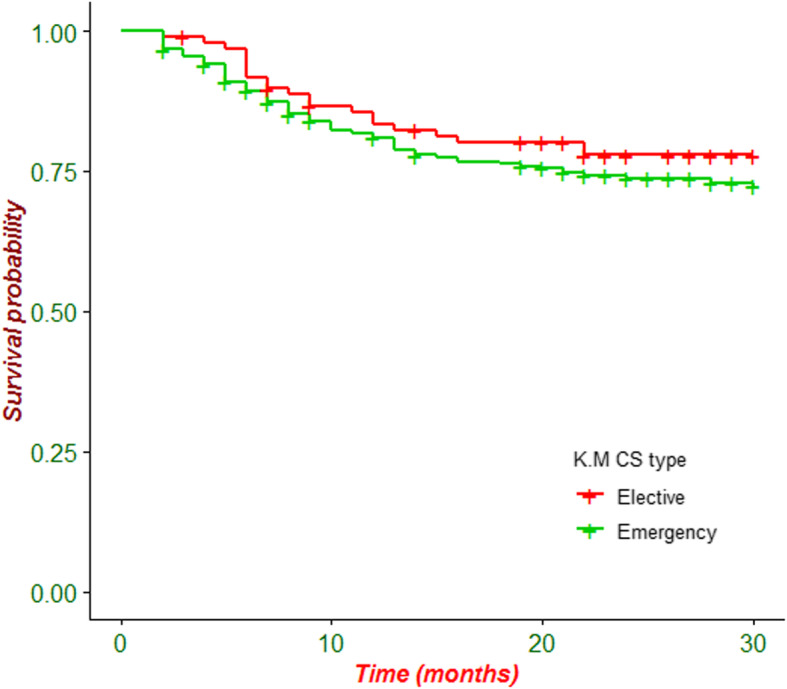
Kaplan-Meier survival curve of patients undergoing caesarean section in Debre Markos Referral Hospital between March 28, 2019 and August 31, 2019 by Type of Surgical Procedure

During the study period, 132 (25.4%) patients had developed SSI with an incidence rate of 11.7 per 1000 (95% CI: 9.8, 13.9) persons/days. Among patients who had develop SSI, about 111(84%) had emergency CS procedure. The rate of SSI was 37/132 (28%) for patients who had rupture of membrane prior to CS procedure. Majority of study participants who had horizontal skin incision experienced SSI (124/132; 93.9%). Patients with SSI had longer hospital stays than those without a SSI (12.7 ± 6.9 days’ vs 4 ± 1.7 days). Due to the high prevalence of censoring in this cohort the median survival time could not be estimated. As indicated in Fig. 1, the incidence of SSI following CS is not statistically significant difference between the urban and rural residents. The survival time was different based on the surgical procedures performed (elective or emergency) (Fig. 2).

### Predictors of incidence of surgical site infections

The multivariable Cox analysis documented that women who are not able to read and write were 1.32 times more likely to develop SSI compared to their literate counterparts (AHR = 1.30, 95% CI, 1.19, 2.11). The incidence of SSI among women who did not have antenatal follow up during pregnancy is 2-folds higher risk relative to those who had antenatal follow up (AHR = 2.16, 95% CI: 1.05, 4.53). Women who had previous history of CS were 21% more risk for SSI (AHR = 1.21, 95%CI: 1.11, 2.31). In our study, the incidence of SSI following CS was significantly different between HIV positive and HIV negative women. The incidence of SSI among HIV positive women is 39% higher compared to HIV negative women (AHR = 1.39,95% CI: 1.21, 2.57). The details of bivariable and multivariable output is presented in Table 5.

**Table 5:** Bivariable and multivariable analysis to identify independent predictors of surgical site infection of patients undergoing caesarean section in Debre Markos Referral Hospital between March 28, 2019 and August 31, 2019

Variables	SSI status		CHR(95% CI)	AHR(95% CI)
	Event	Censored		
**Residence**				
Urban	96	247	1	1
Rural	36	141	0.73 (0.49,1.06)	0.80 (0.51, 1.30)
**Educational Status**				
Not able to read and write	36	145	1.32 (1.21, 3.20)	1.30 (1.19, 2.11)
Primary and secondary	52	145	0.81 (0.71, 1.89)	0.79 (0.89, 2.88)
Tertiary and above	44	96	1	1
**Occupation**				
House wife	70	248	1	1
Merchant	23	44	1.56 (0.97, 2.51)	1.45 (0.90, 2.47)
Employed	39	96	1.30 (0.88, 1.93)	1.07 (0.67, 1.78)
**ANC follow up**				
Yes	122	366	1	1
No	10	22	1.38 (1.22, 2.64)	2.16 (1.05, 4.53)
**CS history**				
Yes	25	82	1.19 (1.12, 1.96)	1.21 (1.11, 2.31)
No	107	306	1	1
**Onset of labor**				
Spontaneous	105	295	1	1
Induced	7	10	1.51 (0.70, 3.20)	1.15 (0.51, 2.64)
No labor	20	83	0.68 (0.42, 1.10)	0.59 (0.27, 1.23)
**Hypertension**				
Yes	10	29	1.03 (0.54, 1.97)	1.11 (0.55, 2.21)
No	122	359	1	1
**HIV/AIDS**				
Yes	2	5	1.16 (1.13, 3.48)	1.39 (1.21, 2.57)
No	130	385	1	1
**CS type**				
Elective	21	77	1	1
Emergency	111	311	1.31 (1.27, 2.03)	1.13 (1.11, 2.43)
**Incision type**				
Horizontal	124	374	1	1
Vertical	8	14	1.74 (1.23, 3.55)	2.60 (1.05, 6.44)
**Skin suturing**				
Interrupted	7	30	1	1
Subcuticular	125	358	1.47 (0.68, 3.15)	2.51 (0.99, 6.30)
**Anesthetic technique**				
General	8	16	1	1
Spinal	124	372	0.70 (0.34, 1.44)	0.69 (0.29, 1.60)
**Rupture of membrane**				
Yes	37	121	1.53 (1.12, 1.65)	1.50 (1.31, 1.64)
No	95	267	1	1
**Frequency of PV**				
Not done	27	92	1	1
1–6	61	203	1.04 (0.66, 1.64)	0.78 (0.38, 1.57)
≥ 7	44	93	1.57 (1.43, 2.46)	1.88 (1.71, 3.20)

## Discussions

This study was designed to determine the incidence of SSI of patients underwent CS in Debre Markos Referral Hospital. SSI is remained one of the major causes of maternal morbidity and mortality after CS delivery [34, 35]. In this study, the overall cumulative incidence of SSI following CS was 25.4% with an incidence rate of 11.7 (95% CI: 9.8, 13.9) per 1000 persons/days. Previous studies conducted in Ethiopia [18, 19], and Estonia University Hospital [36] have documented similar findings. However, the incidence of SSI following CS in the present study was higher compared to studies from developed countries: Oman (2.66%) [37], United States of America (5%) [9], Norway (8.3%) [38], and United Kingdom (9.6%) [39]. This discrepancy could be explained by the standard of hygiene practiced in developed countries and/or by the lack of infection prevention policies in developing countries.

This study revealed various risk factors which predict the incidence of SSI following CS. Prolonged rupture of membranes and multiple vaginal examinations were significant predictors of SSI in this study, which align with previously obtained results [9, 23, 40, 41]. Normally during pregnancy, cervical mucus plug, fetal membranes, and amniotic fluid all serve as barriers to infection [42]. However, when these natural barriers are disturbed, this protective effect is interrupted as amniotic fluid loses its sterility. It is thought that the non-sterile amniotic fluid may act as a transport medium by which bacteria come into contact with the uterine and skin incisions potentiating chorioamnionitis and its sequelae.

Likewise, this study showed that women who are not able to read and write have increased risk of SSI following CS delivery. This finding was similar to findings in previously published studies [21, 24, 43]. This finding could be due to more educated mothers having healthier reproductive practices because they have better knowledge about health care and nutrition, healthier behaviors, and access to more sanitary and safer environments for their health [44]. A mother’s education is widely suggested to positively affect her own and her children’s health and nutrition in developing countries.

In this study, emergency surgery predisposes women to development of SSI as compared to elective procedure. Hospitals with a strict policy on reducing primary CS may go for a decision on CS only after a trial of labor. As a result, most of the CS procedures were performed as an emergency, even if the indication for CS was present in advance. This trend could be explained by improper counselling of pregnant women necessitating CS, causing a delay in hospital attendance. These emergency surgeries have a higher chance of SSI [23, 45]. A similar study conducted in Ethiopia showed that emergency surgery had two times increased risk of surgical site infection compared to elective cases [19]. This finding could be attributable to the fact that, in emergency cases, membrane rupture and multiple vaginal examinations are more frequent. There is also increased risk of bacterial contamination, breaks in sterile technique, and/or lack of timely antibiotic prophylaxis.

In our study, an increased rate of SSI was observed in women who experienced vertical incisions. This finding was in agreement with studies conducted in India [46] and Tanzania [24]. This finding could be related to the procedural elements as it takes longer for vertical incisions thereby at risk for more frequent contact and increased chance of contamination. In our study women who did not have antenatal care (ANC) follow up during pregnancy had 2 fold increased risk for SSI compared to women who had ANC follow up. This finding was supported through several studies [19, 27, 29]. Since ANC provides women and their families with appropriate information and advice for a healthy pregnancy, safe childbirth, and postnatal recovery, the decrease in incidence of SSI infection following CS may be the outcome of this proactive intervention.

### Limitations of the study

Our study has limitations. Some of the cases during the study period were not followed up due lost to follow up which could likely influence the calculated rate of surgical site infection. Further the study only occurred in a single hospital which may have a fairly homogenous catchment of women. Additionally, the DMRH is a centrally based hospital receiving clients what are likely at a higher risk than the population at large, which limits the extrapolation of the findings beyond similar contexts. In addition, microbiological isolation was not conducted in this study.

## Conclusions

This study concludes that the incidence of surgical site infection following caesarean section was relatively high compared to previously published studies. Significant predictors of SSI in this study included: women who were not educated, did not have ANC follow-up, had previous history of CS, were HIV positive, and/or encountered emergency surgery, vertical incision, rupture of membranes before CS, and multiple vaginal examinations. Therefore, increased awareness on these risk factors informing development and strict implementation of protocols may minimize and prevent the high SSI rate after caesarean section. In addition, providing health education of patients and advise on preventing surgical site infections before and after surgery should be considered.

## Supplementary Information

**Additional file 1:** **Table S1.** Scaled Schoenfeld residuals tests of proportional hazard assumption.

**Additional file 2:** **Figure S1.** Plots of scaled Schoenfeld residuals against transformed time for the model.

**Additional file 3.**

## Data Availability

The datasets used and/or analyzed during the current study are available from the corresponding author on reasonable request.

## Abbreviations

CS: Caesarean section
CI: Confidence Interval
HIA: Healthcare-Associated Infection
HR: Hazard Ratio
WHO: World Health Organization
MMR: Maternal Mortality Ratio
ANC: Antenatal Care
KM: Kaplan-Meier
SSI: Surgical Site Infection
HIV: Human Immune Deficiency Virus
PV: Peravaginal examination
IQR: Inter Quartile Range
