# Supplementary material for: Incidence and predictors of surgical site infection following cesarean section in North-west Ethiopia: a prospective cohort study
Source: BMC Infect Dis. 2020 Nov 30;20:902. doi: 10.1186/s12879-020-05640-0 (PMC7708170; doi:10.1186/s12879-020-05640-0)
Supplement: Supplementary file 1 — Additional file 1: Table S1. Scaled Schoenfeld residuals tests of proportional hazard assumption. [file 12879_2020_5640_MOESM1_ESM.docx]

| **Variables** | **Rho** | **Chisq** | **P** |
| --- | --- | --- | --- |
| **Resident** |  |  |  |
| Urban | 0.09 | 1.13 | 0.287 |
| **Education** |  |  |  |
| Primary or secondary | 0.016 | -0.00361 | 0.849 |
| Tertiary and above | 0.019 | -0.00504 | 0.822 |
| **Occupation** |  |  |  |
| Merchant | 0.139 | 2.63 | 0.105 |
| Employed | 0.09 | 1.37 | 0.241 |
| **ANC follow up** |  |  |  |
| No | -0.103 | 1.51 | 0.220 |
| **Hypertension** |  |  |  |
| Yes | -0.031 | 0.144 | 0.705 |
| **CS type** |  |  |  |
| Emergency | 0.0004 | 0.00000276 | 0.999 |
| **Skin suturing type** |  |  |  |
| Subcuticular | -0.066 | 0.7760.378 |  |
| **Anesthetic type** |  |  |  |
| Spinal | -0.046 | 0.373 | 0.54 |
| **Number of Per viginal examination** |  |  |  |
| 1-6 | -0.0257 | 0.00797 | 0.778 |
| >=7 | 0.0255 | 0.00854 | 0.770 |
| **Rupture of membrane before CS** |  |  |  |
| Yes | 0.0118 | 1.8 | 0.18 |
| Global test | NA | 12.1 | **0.519** |
