# Supplementary material for: Incidence and predictors of surgical site infection following cesarean section in North-west Ethiopia: a prospective cohort study
Source: BMC Infect Dis. 2020 Nov 30;20:902. doi: 10.1186/s12879-020-05640-0 (PMC7708170; doi:10.1186/s12879-020-05640-0)
Supplement: Supplementary file 2 — Additional file 2: Figure S1. Plots of scaled Schoenfeld residuals against transformed time for the model. [file 12879_2020_5640_MOESM2_ESM.docx]

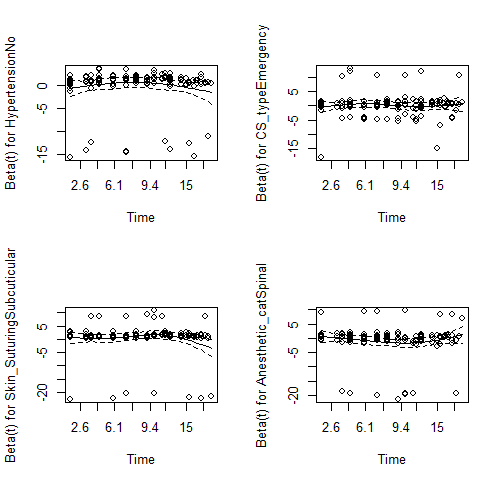


Fig 1: Plots of scaled Schoenfeld residuals against transformed time for variables in a model
fit to the data
