# Supplementary material for: Incidence and predictors of surgical site infection following cesarean section in North-west Ethiopia: a prospective cohort study
Source: BMC Infect Dis. 2020 Nov 30;20:902. doi: 10.1186/s12879-020-05640-0 (PMC7708170; doi:10.1186/s12879-020-05640-0)
Supplement: Supplementary file 3 — Additional file 3. [file 12879_2020_5640_MOESM3_ESM.pdf]

Title: Incidence and predictors of surgical site infection following caesarian section

**Patient's MRN.---------------------------------**			
**No**	**Variable**	**Coding categories**	**Remark**
101	Patients ID Number		
102	Age in year	________________________	
104	Residence	1. Urban 2. Rural	
105	Religion	1. Orthodox 2. Muslim 4. Protestant 4. Catholic<br>5. Other (please specify___________________________)	
106	Occupation	1. House wife 2. Merchant 3. Employee 4. Student<br>5. Other specify ------------------	
107	Education	1. Not educated 2. Able to read and write 3. Primary<br>4. Secondary 5. Tertiary and above	
108	Antenatal check up	1. Yes 2. No	If no skip to 112
109	Place of ANC follow up	1. DMRH 2. Outside	
110	If outside where? Specify	__________________	
111	Number of prenatal care visits	___________________	
112	Previous CS	1. Yes 2. No	If no skip to 114
113	If yes for Question no.112 number of prior CS	_____________________	
114	Parity	_____________________	
115	Pre-surgery hospital stay	_____________________	
116	Onset of labor	1. Spontaneous 2. Induced 3. No labor	
**Section II :Comorbidities (Medical history)**			
**No**	**Variable**	**Coding categories**	**Remark**
201	Hypertension (any type)	1. Yes 2. No	
202	Diabetes mellitus	1. Yes 2. No	
203	Renal disease	1. Yes 2. No	
204	Bronchial asthma	1. Yes 2. No	
205	Thyroid dysfunctions	1. Yes 2. No	
206	HIV	1. Positive 2. Negative 3. Unknown	
207	Hepatitis B	1. Yes 2. No 3. Unknown	
208	Arthritis	1. Yes 2. No	
209	systemic steroid use	1. Yes 2. No	
210	Anemia	1. Yes 2. No	
**Section III: Operational related variables**			
**No**	**Variable**	**Coding categories**	**Remark**
300	Date of admission dd/mm/yy	___________________	
301	Rapture of membrane before CS	1. Yes 2. No	
302	If rupture duration of ROM in hrs.	____________________	In hour
303	Per virginal examination	1. Not done 2. Done (how many) specify ______	

304	Preoperative blood transfusion	1. Yes 2. No	
305	Date of CS done dd/mm/yy	______________________	
306	Start time of CS hr/mm/second	______________________	
307	End time of CS hr/mm/second	______________________	
308	Duration of procedure	______________________	In minute
309	Types of CS	1. Elective 2. Emergency	
310	Types of incision	1. Horizontal 2. Vertical	
311	Surgical wound class	1. Clean 2. Clean contaminated<br>2. Contaminated 3. Dirty/infected	
312	Types of skin suturing	1. Interrupted<br>2. Subcuticular	
313	Number of people in theatre	______________________	
314	Estimated of blood loss	________________________ml	
315	Maternal weight	_________________________kg	
316	Anesthetic techniques	1. General 2, Epidural 3. Spinal	
317	Indication for CS	1. Fetal distress 2. Previous CS 3. Breech presentation<br>4 Poor progress 5. Placenta Previa type 3<br>6 Severe macrosomia 7 Twin babies<br>8 Sever preeclampsia 9 Other(Specify)___________	
319	Gestational age	_____________________wks	In week
320	Hemoglobin previous to cesarean	____________________g/dL	
321	Hemoglobin after CS (g/dL)	___________________g/dL	
320	Leukocyte count previous to cesarean (cells/μL)	____________________cells/μL	
321	Indwelling urethral catheter	1. Yes 2. No	
322	Soap and water bath before surgery	1. Yes 2. No	
324	Blood transfusion	1. No transfusion 2. Transfused ------------- units	
325	Pre-operative waiting period	___________________days	
326	Prophylactic antibiotic	1. Yes 2. No	
327	Procedure performed by	1. Gynecologist 2. Emergency surgeon<br>3 Junior surgeon 4. Other (Specify)------------------	
329	Child alive	1. Yes 2. No	
330	Sign of inflammation<br>1. At first visit ---------------<br>2. Second visit ---------------<br>3. Third visit ------------------<br>4. Forth visit -------------------	1. No sign of inflammation<br>2. Induration<br>3. Local site of fever<br>4. Erythema<br>5. Purulent discharge<br>6. localized swelling<br>7. dehiscence, overlying skin changes<br>8. wound deliberately opened by the surgeon for drainage<br>9. Local site of fever	
331	Date of any sign observed dd/mm/yy	----------------------	
332	Date of discharge dd/mm/yy	-------------------	
**4**	**Regarding surgical antimicrobial prophylaxis used,**		

400	place at which antimicrobials were administered	1. Ward 2. Operation room 3. Both	
401	generic name of antimicrobials	1. --------------------------------------<br>2. --------------------------------------<br>3. ---------------------------------------<br>4. ---------------------------------------<br>5. --	
402	Time of first dose administration	----------------------------------	
403	Route of administration		
404	Time of intra-operative dose administration	-----------------------	
405	time of postoperative dose given and duration of administration	--------------------------------------	

Sign of inflammation at least one of the following at surgical site: **Induration, Local site of fever, Erythema, Purulent discharge, Organisms isolated from fluid, localized swelling, dehiscence, overlying skin changes, wound deliberately opened by the surgeon for drainage, Surgeon declares that the wound is infected**

**Address**	**1**	**2**	**3**
**Date of appointment for phone call**			
